# Development and validation of MyCommunication-Youth: A self-report measure for communicative participation in children, adolescents and young adults

**DOI:** 10.1186/s41687-025-00913-1

**Published:** 2025-07-10

**Authors:** Eline Alons, Lizet van Ewijk, Margreet Roelien Luinge, Nicole ter Wal, Tonny Methorst, Ellen Gerrits, Caroline Barbara Terwee

**Affiliations:** 1 Research Centre Healthy and Sustainable Living, HU University of Applied Sciences, Utrecht, The Netherlands; 2 Department of Languages, Literature and Communication, Institute for Language Sciences (ILS), Utrecht University, Utrecht, The Netherlands; 3 Research Centre Healthy Ageing; Youth, Education and Society, Hanze University of Applied Sciences Groningen, Groningen, The Netherlands; 4 Amsterdam UMC location Vrije Universiteit, Epidemiology and Data Science, Amsterdam, The Netherlands; 5 Amsterdam Public Health Research Institute, Methodology, Amsterdam, The Netherlands

**Keywords:** Children, Adolescents, Young adults, Participation, Outcome, Content validity

## Abstract

**Background:**

Communicative participation is the most important outcome of speech and language therapy, but there are no measurement instruments for children, adolescents, and young adults. This paper describes the development of MyCommunication-Youth: an item bank to measure self-reported communicative participation in children, adolescents and young adults with various communication disorders.

**Aims:**

1) To develop a comprehensive, comprehensible item bank for measuring communicative participation in children, adolescents, and young adults with communication problems. 2) To assess the content validity of the item bank in a sample of these groups. 3) To define criteria for the minimal age at which children with communication difficulties can self-report their communicative participation.

**Method:**

Based on a literature review and two concept elicitation studies three initial versions of item pools were developed: one for children, one for adolescents and one for young adults. These pools were pilot tested, using cognitive debriefing interviews, on comprehensibility and comprehensiveness in a diverse group of participants with communication difficulties, whereafter a second version of the item pools were created. Hereafter, the content validity was assessed in the target population and in a group of speech and language therapists.

**Results:**

Initially, three item pools were created for children (58 items), adolescents (78 items), and young adults (84 items). In the pilot test with 33 children adolescents and young adults with communication difficulties, items were revised for comprehensibility, some items were added for comprehensiveness, and some items were deleted because they appeared irrelevant, resulting in updated pools of 50, 69, and 72 items. In the content validity study, cognitive interviews with 27 participants and a focus group with 8 professionals identified additional revisions. Most items were comprehensible, but some were irrelevant for specific subpopulations of communication difficulties. Two new items were added after the input of professionals, whereafter the item bank was found comprehensive.

**Conclusions:**

MyCommunication-Youth is an item bank for measuring communicative participation in children, adolescents and young adults with various communication difficulties. Three versions of the instrument were created: MyCommunication-Children of 49 items, MyCommunication-Adolescents of 70 items and MyCommunication-YoungAdults of 73 items. The item bank is comprehensible, relevant and comprehensive according to the target population and target professionals.

**Supplementary information:**

The online version contains supplementary material available at 10.1186/s41687-025-00913-1.

## Introduction

The measurement of patient-reported outcomes (PROs) has become increasingly important in healthcare for shared decision-making and value-based healthcare [1, 2]. PROs ensure that clients’ perspectives are taken into account in determining treatment needs, selecting and prioritizing treatment goals, and evaluating whether meaningful outcomes have been achieved [3]. Applied to the field of speech and language therapy, person-centered care requires speech and language therapists (SLTs) to capture each client’s perspective on their communication problems and to document that the interventions have resulted in meaningful changes that clients define as worthwhile [4, 5]. Meaningful change within speech and language therapy is often referred to as changes in life situations that require communication, also known as communicative participation [6].

Communicative participation is defined as “Taking part in life situations where knowledge, information, ideas or feelings are exchanged” [6]. It is considered to be the most important outcome of speech and language therapy [7, 8, 9, 10], relevant for all individuals with communication difficulties, regardless of the nature and severity of their problems [6, 8]. Given that it is the most important outcome, it is essential to measure communicative participation. Although there are many PRO measures (PROMs) within the field of speech and language therapy, for both children and adults [11, 12], there are only few PROMs specifically targeted at communicative participation.

The Communicative Participation Item Bank [13] and MyCommunication-Adults [14] are two examples of PROMs that capture communicative participation from the perspective of adults with communication difficulties. These PROMs are based on item banks, which are basically collections of large numbers of questions, all addressing one construct [15]. The characteristics of the items in an item bank (difficulty and discrimination) are determined by item response theory (IRT). Based on the information on the item characteristics and the purpose of measuring, different types of PROMs can be generated from an item bank. For instance, different short forms comprising a selected number of highly informative questions may be generated. Alternatively, computer adaptive tests may be employed, whereby the computer determines the subsequent question based on answers to the questions already administered.

In a systematic review of PROMs and parent reports [12], only one instrument was identified that measures communicative participation in children: the Focus on Outcomes of Communication Under Six (FOCUS) [16]. The FOCUS is a parent-reported questionnaire for parents of children with communication difficulties up to 6 years old. PROMs for measuring communicative participation do not exist [7, 12], which means that we are not able to measure the key outcome of speech and language therapy in children and adolescents. In the development and validation of PROMs on communicative participation for adults, participants were eligible from the age of 18 [13, 14]. However, no young adults took part in the concept elicitation phase. During the content validity assessment of MyCommunication-Adults, two young adults participated and noted that relevant items reflecting their life context were missing, consisting of situations involving gaming and participation in school [14]. These contexts are specifically related to the stage of life that young adults are in, so it would be beneficial to have an instrument for this age group as well.

The use of self-report instruments for children’s perspectives is not yet common in speech and language therapy [12, 17]. Studies in other fields show children can report on their health using self-report questionnaires [18, 19, 20], but determining the exact age for accurate self-reporting varies by population, context, and construct [21]. Age boundaries are challenging due to variations in motor, cognitive, and linguistic abilities among children [22]. Some authors suggest children as young as five can self-report [19, 20], while others place the cut-off between six and eight years [21]. For children with communication disorders, this is even more complex due to their linguistic challenges and the abstract nature of communicative participation compared to more concrete constructs like pain or itching.

In conclusion, communicative participation is the most important outcome of speech and language therapy, but there are no measurement instruments for children, adolescents, and young adults. This paper describes the development of an item bank to measure communicative participation in these groups: MyCommunication-Youth, an instrument in line with MyCommunication-Adults [14]. The study aims to: 1) Develop a comprehensive, comprehensible item bank for measuring communicative participation in children, adolescents, and young adults with communication problems. 2) Assess the content validity of the item bank in a sample of these groups. 3) Define criteria for the minimal age at which children with communication difficulties can self-report their communicative participation.

## Method

This study describes the development of MyCommunication-Youth and describes a combination of qualitative cognitive interviews with children, adolescents and young adults and a focus group session with SLTs. Data was collected between July 2023 and October 2024. For the development of MyCommunication-Youth, the standards of the COnsensus-based Standards for the selection of health Measurement INstruments (COSMIN) initiative were used [23–25]. The study was approved by the Internal Review Board of the HU University of Applied Sciences Utrecht (reference number 241-001-2023) and was conducted according to the principles of the Declaration of Helsinki and in accordance with the Dutch Medical Research Involving Human Subjects Act (WMO).

### Clarification of construct: Clear description and origin

Communicative participation was defined as “taking part in life situations where knowledge, information, ideas or feelings are exchanged. It may take the form of speaking, listening, reading, writing and nonverbal means of communication”[6]. The definition contains a combination of two separate constructs: 1) communication (the exchange of knowledge, information, ideas, or feelings) and 2) participation (taking part in a life situation). Communicative participation is about the interaction (i.e. a message with the opportunity for a response) between two persons or more, in the context of a life situation [6].

For the development of MyCommunication-Youth, the definition of the construct of communicative participation was further operationalized, in collaboration with the authors of the development of the item bank for adults: MyCommunication-Adults [14]. This operationalization entails considerations for communication and considerations for participation.

For the interpretation of communication (the exchange of knowledge, information, ideas, or feelings), we considered “exchange” as “an exchange with the opportunity for a direct, prolonged, or behavioral response between two communicative partners with the purpose of establishing joint meaning”. For the interpretation of participation (taking part in life situations) we chose to interpret “life situations” as “the performance of social roles in the ICF Activities and Participation domains *mobility, self-care, domestic life, interpersonal interactions and relationships, major life areas* and *community social and civic life*” (Domain 4–9 [26]).

### Target population

MyCommunication-Youth is designed to measure self-perceived communicative participation in children, adolescents and young adults with various communication problems. This includes speech disorders, language disorders, voice disorders and hearing loss.

### Context of use

MyCommunication-Youth was developed for clinical practice with three purposes:

1. To identify self-perceived communicative participation problems in children, adolescents and young adults with communication difficulties;
2. To support patient-centered care by including the patients’ perspectives;
3. To evaluate the effects of therapy on the most important outcome of SLT; communicative participation.

In addition, MyCommunication-Youth can be used as an outcome measure in clinical studies.

### Development

**Fig. 1:**
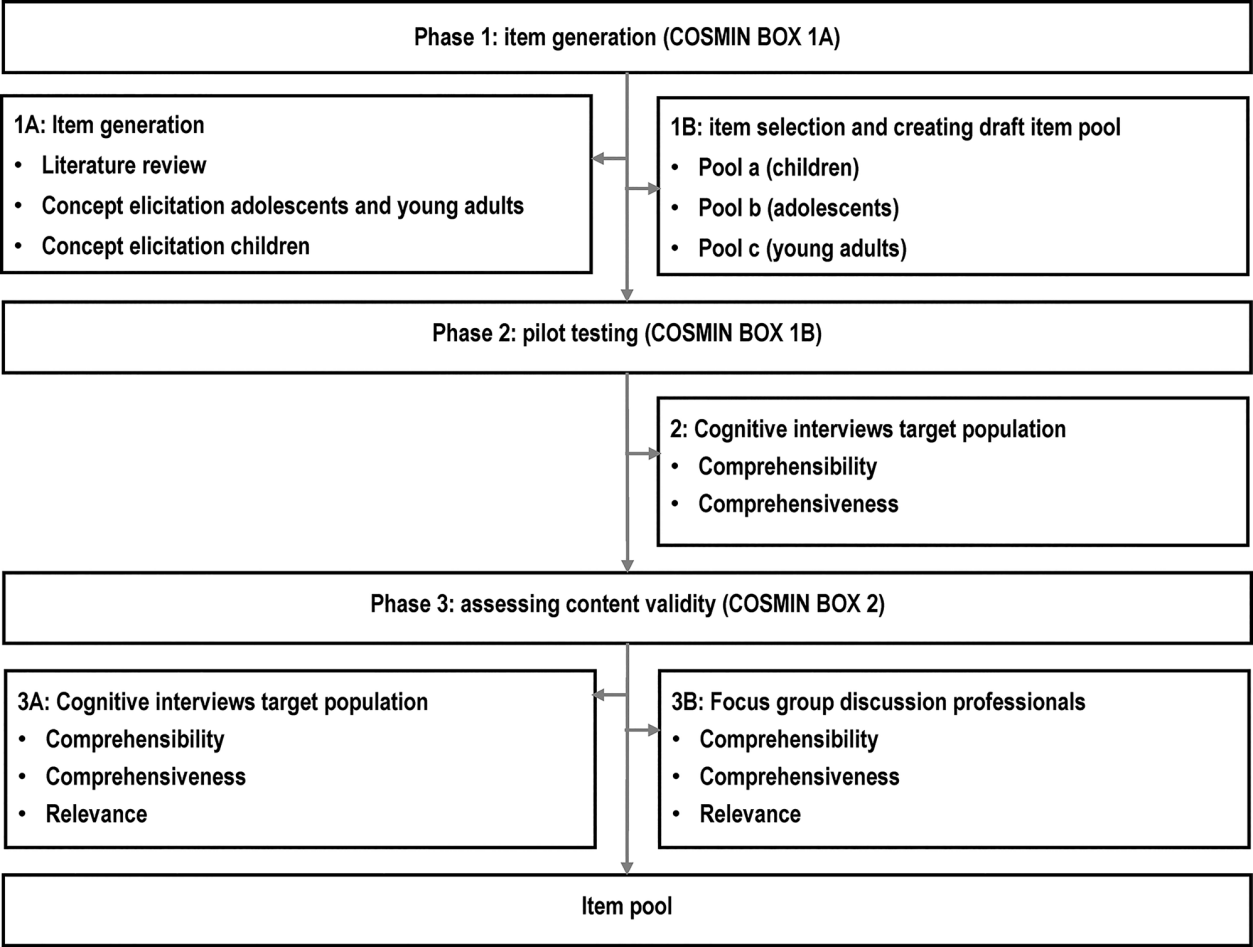
Overview of phases and steps development MyCommunication-Youth

The development of the MyCommunication-Youth consists of several steps, that are divided into three phases. Figure 1 presents an overview of all the steps.

### Phase 1: Item generation

This phase contains several steps. Phase 1a comprised three sub-studies that have been published separately. Below we describe a brief summary of these studies. Phase 1b and Phase 2 and 3 are reported in this paper.

### Item generation

#### Literature review [12]

The purpose of the literature review was to select existing items that measure communicative participation from PROMs and parent reports that measure (aspects of) communication or participation in children and adolescents with communication disorders. A systematic search was performed in EMBASE and PubMed to find existing measurement instruments. 29 instruments were found, 20 parent reports and 9 PROMs. 145 out of a total of 1149 items were identified that measure communicative participation. These items were used as input for writing new items for our item bank.

#### Concept elicitation studies

In two concept elicitation studies, the aim was to identify relevant, self-experienced situations in which children [27], adolescents and young adults [28] have difficulties participating because of communication problems. Participants were asked to keep a diary in their own preferred way, in which they kept situations that occurred during that day. During a subsequent interview, these situations were further explored. A total of 405 situations were discussed (234 situations by adolescents and young adults, 171 situations by children) The situations were used to write items in phase 1b.

### Item selection and creating draft item pool

The 145 items from the literature review and 405 items from the concept elicitation studies were combined. Duplicates were removed. The research group established criteria that the items had to meet:

- Positive wording of the items (item stem: I can …) and asking about current capabilities, making the recall period irrelevant;
- Response format consistent with MyCommunication-Adults [14]
  - 4-point scale: with no difficulty, with a little difficulty, with much difficulty, cannot do.
  - Follow-up question when the answer is with a little difficulty or cannot do: 1) I don’t mind, 2) I mind a little, 3) I mind a lot.
- Language communication friendly using the *Guidelines for communication-friendly questionnaires* [29], e.g.
  - Dutch language level B1 or lower;
  - Use of concrete words;
  - Short sentences of a maximum of 8–12 words.

We followed the Guideline for Communication-Friendly Questionnaires [29], which covers five main topics: 1) visualizations, 2) language representation, 3) language meaning, 4) answer scales, and 5) structure. This study focused on topics 2–5, while visualizations were not included. The item bank is designed for people with and without language comprehension difficulties. Our goal was to create a general item pool using plain language suitable for most of the target population, with the potential to add visual aids later for those who struggle with written language.

Two researchers (EA and NW, both SLT, female, and PhD students) removed duplicates and rewrote items, followed by team discussions. Further revisions were made by EA and CT (expertise in PROMs) to meet the guideline criteria. We discussed that some items were only appropriate in specific life stages (e.g. items about play or work). We therefore decided to create three versions of MyCommunication-Youth; one for children, one for adolescents, and one for young adults.

### Phase 2: Pilot testing

The aim of the pilot test was to evaluate whether each draft item pool was comprehensive and understandable for the target population. According to COSMIN, comprehensibility means the items are understood as intended, and comprehensiveness means no key concepts are missing [25]. This was assessed through cognitive interviews, where participants reviewed instructions, items, and response options. To evaluate comprehensibility, participants explained the instructions and responses in their own words, and for the items, they selected an answer and explained their choice. This process helped verify whether participants understood the material as intended. To facilitate this process, a list of examples of intended meanings was created (see Supplementary Materials 2).

To minimize participant burden, each reviewed about half of the items. Items were shown by theme, and after reviewing a theme, participants were asked if any key concepts were missing. This method allowed for an assessment of comprehensiveness, even though the entire item pool was not reviewed. The interview guide is provided in Appendix 1 (all appendices are included in Supplementary Materials 1).

A diverse group of children, adolescents, and young adults (ages 5–25) with various communication challenges was recruited, using a purposive sampling strategy. Participants were recruited in three ways: 1) though messages via the Dutch Association of Speech and Language Therapy (NVLF); 2) social media (LinkedIn, Facebook pages for SLTs or parents); 3) network of the researcher.

In the Netherlands, specific consent regulations apply to research participation based on age. For children under 12 years old, only parental consent is required. For participants aged 12–16, both the child and their parents must provide consent. From the age of 16, participants can provide consent independently. To ensure clear communication, three versions of information letters were created: 1) A letter for parents; 2) A letter for children aged 12–16, designed to be age-appropriate and supported with images for visual information. 3) A letter for adolescents and young adults (>16 years) with standard wording. 4) A letter for adolescents and young adults with language difficulties, using adjusted wording and visual support with images. Additionally, an information video was available to explain the study’s purpose and what was expected of participants. Parents were encouraged to watch the video with their child, which could be accessed via a QR-code. Written informed consent was obtained from all participants and/or their parents before participation in the study.

Interviews were conducted by the first author (EA), at a location of the participant’s choice, which was often at home and in some cases at school. If preferred by the participant, parents were allowed to be present during the interview. EA had prior experience conducting qualitative research. All interviews were audio-recorded and transcribed verbatim, followed by content analysis. After every two interviews, results were analyzed and revisions were made to the instructions, response options, and items. If changes seemed relevant for other subgroups, those items were also revised. This iterative process continued until each item was deemed comprehensible by at least four participants in each subgroup. The result was the second version of the draft item pool.

### Phase 3: Assessing content validity

#### Focus group with professionals

In addition to cognitive interviews with the target population, a focus group with professionals was conducted to assess the comprehensibility, comprehensiveness, and relevance of the second version of the item pool [25]. Seven speech-language therapists (SLTs) with expertise in children, adolescents, young adults, and communication disorders (speech, language, hearing, or voice) were recruited, along with one senior and one junior researcher with experience working with the target population.

The participation process involved two steps. First, participants completed a preparation task, rating the entire item pool on seven criteria (see Table 6). These ratings were analyzed by the first author (EA). Items receiving less than 85% consensus were flagged for discussion. In the second step, a focus group meeting was held to discuss items with low consensus, aiming to reach an agreement on necessary adjustments, removals, or additions. The session was facilitated by EA and TM (SLT and junior researcher), and audio recorded.

Results were analyzed and discussed with three researchers (EA, ML; senior researcher and neurolinguist, CT). The ratings of the participants were converted into percentages for relevance and comprehensibility. If items were missing, new items were written, whereafter they were pilot-tested and assessed on content validity by the target group before they were definitively added to the item pool.

#### Defining age boundaries

As it is unclear at what age children can accurately report on communicative participation using questionnaires, we included children as young as 5 years old to assess whether they understood the items, instructions, and response options, and could select an appropriate response. We evaluated their ability to respond using the following criteria:

For comprehensibility of the items we distinguished four categories: 1) The child can explain the meaning of items 2) The child cannot explain the meaning of items, but the researcher observes understanding; 3) The child cannot explain the meaning of items, the researcher observes partial understanding; 4) The child cannot explain the meaning of items, and the researcher observes no understanding. For comprehensibility of the response options, we distinguished three categories: 1) Understood, explained by the child; 2) Understood, as observed by the researcher; and 3) Not understood. The ability to reflect upon comprehensiveness was assessed using two categories: 1) Yes and 2) No.

**Fig. 2:**
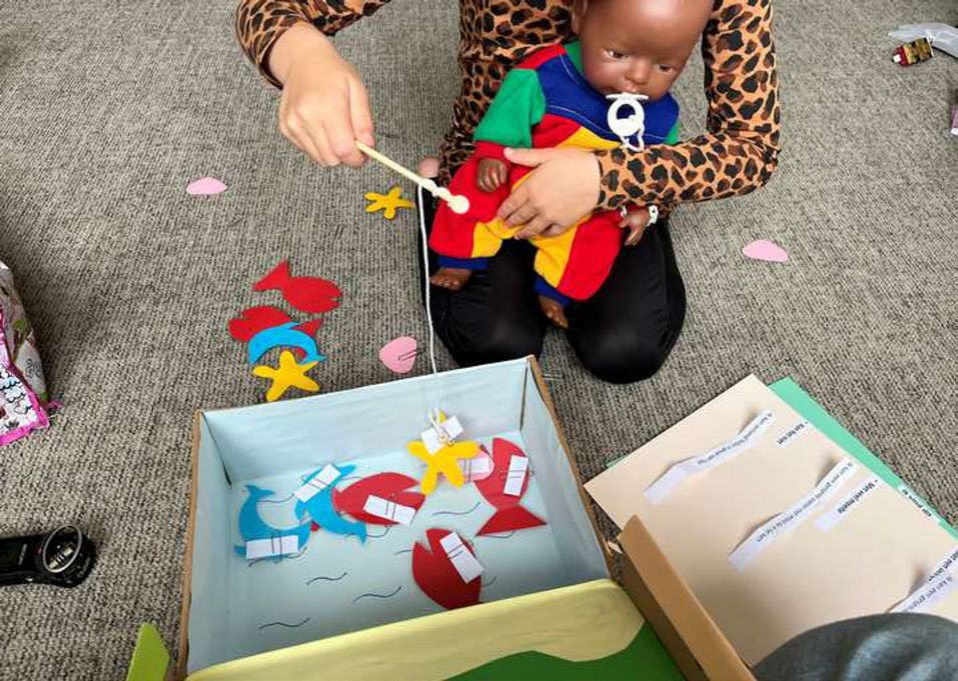
Playful set-up for cognitive debriefing interviews children

Instructions, items, and response options were shown one by one using Microsoft PowerPoint. For the youngest participants, we created an age-appropriate, playful setting as recommended by Gale & Carlton [30]. We used a “pool” with little fish that had items on their backs. Children fished out the items, answered them using a paper response format, and then answered questions about comprehensibility and comprehensiveness. Figure 2 shows a photo of this setup.

### Cognitive interviews in the target population

This study aimed to assess the content validity of the second draft of the item pool in the target population. This study was performed in a different sample of people with communication disorders than in the pilot study. Interviews were conducted by the first author (EA). The process of recruitment and informed consent was similar to the pilot study.

Comprehensibility and comprehensiveness were assessed using the same method as in the pilot test. In addition, relevance was tested (i.e. all items should be relevant for the target population [25];). As relevance is a difficult word, it was asked using the following questions: *is this item important to ask to other* [children/adolescents/youngadults] *with* [name communication problem]*?* The interview guide for this study is provided in Appendix 2.

All interviews were audio-recorded and transcribed verbatim. The results were analyzed by EA and NW together. The percentage of items that were found comprehensible and relevant to the target population was calculated.

## Results

### Phase 1: Item generation

The item generation resulted in three draft versions of MyCommunication-Youth: pool (a) for children of primary school age (approximately 5–12) with 58 items, pool (b) for adolescents high school age (approximately 13–18) with 78 items and pool (c) for young adults, working or studying (approximately 19–25) with 84 items.

### Phase 2: Pilot testing

Thirty-three participants participated (Table 1).

**Table 1:** Participants with communication difficulties in pilot test

Pp	Age	Classification	Gender	Communication problem**	Relevant co-morbidities	Help reading?***	Multilinguaism?	Included in item analysis?****
P01	23	YA	F	Speech, language (verbal dyspraxia + DLD)		N	Yes, Dutch/German	Y
P02	25	YA	F	Language (DLD)		N	No	Y
P03*	7	C	F	Hearing		Y	No	Y
P04	9	C	F	Language (DLD) and dyslexia		Y	No	Y
P05	6	C	F	Language (DLD)		Y	No	Y
P06	13	A	M	Language (DLD) and dyslexia		N	No	Y
P07	17	A	M	Speech (stuttering)		N	No	Y
P08*	7	C	F	Speech (CP)	Cerebral Palsy, delayed information processing.	Y	No	N
P09	11	C	F	Speech (stuttering)		N	No	Y
P10	21	YA	M	Hearing (otosclerosis)	ADHD	N	No	Y
P11*	9	C	M	Hearing (unilateral)		N	No	Y
P12*	5	C	M	Hearing (bilateral)		Y	No	N
P13*	13	YA	F	Language (DLD)		N	No	Y
P14	12	C	F	Language (DLD)		N	No	Y
P15	26	YA	M	Language (DLD)	Autism	N	Yes, Dutch/Turkish	Y
P16	27	YA	F	Language (DLD)		N	No	Y
P17	11	C	M	Language (DLD)		N	No	Y
P18	12	C	M	Language (DLD)		N	Yes, Dutch/ English/Arabic	Y
P19	9	C	M	Speech		N	No	Y
P20	8	C	F	Language (DLD)		Y	Yes, Dutch/English	Y
P21	8	C	F	Language (DLD)		Y	Yes, Dutch/arabisch	Y
P22	7	C	M	Speech, Language (DLD, stuttering)		Y	No	N
P23	7	C	F	Language (DLD)		Y	No	N
P24	5	C	F	Language (DLD)		Y	No	N
P25	19	YA	M	Speech (stuttering)		N	No	Y
P26	26	YA	F	Language (DLD)		N	No	Y
P27	22	YA	M	Speech (cleft)		N	No	Y
P28	14	A	M	Speech (stuttering)		N	No	Y
P29	15	A	F	Language (DLD)		N	No	Y
P30	17	A	M	Language (DLD)		N	No	Y
P31	16	A	M	Language (DLD)		N	Yes, Dutch/Polish	Y
P32	17	A	F	Language (DLD)		N	No	Y
P33	17	A	M	Language (DLD)		N	Yes, Dutch/Afghani	Y

*Parent was present during interview

**As reported by participant or parent

***Help reading included reading out loud the entire item or part of the item

****Based on observations of the ability of children to reflect upon communicative participation. If not able, participants were excluded from item analysis

In pool (a) for children, 12 items were revised, 11 items were deleted, and 3 items were added. In pool (b) for adolescents, 11 items were revised, 10 items were deleted, and 1 item was added. In pool (c) for young adults, 19 items were revised, 12 items were deleted, and no items were added. Instructions were revised for all item pools (see Table 2). No changes were made to the response options. An overview of the items before and after the pilot test, including removed items and the reasons for revisions, can be found in the supplemental materials.

**Table 2:** Changes to instructions of PROM

Phase	Instructions (Dutch)	Instructions (English)
Initial version item pool	Communiceren betekent dat je praat, luistert, leest, schrijft of begrijpt. Je krijgt vragen over situaties waarin je moet communiceren. Hoe goed kan jij meedoen in deze situaties? Er is geen goed of fout antwoord.	Communicating means talking, listening, reading, writing or understanding. You will be asked questions about situations in which you need to communicate. How well can you participate in these situations? There is no right or wrong answer.
Second version item pool	Je krijgt vragen over situaties waarin je gaat meedoen. Hoe goed kan jij meedoen? Er is geen goed of fout antwoord.	You will be asked questions about situations in which you will participate. How well can you participate? There is no right or wrong answer.
Final version item pool	Je krijgt vragen over allerlei situaties. - Situaties waarin je moet begrijpen - Situaties waarin je moet praten. Hoe goed kan jij meedoen in deze situaties? Er is geen goed of fout antwoord.	You will be asked questions about different situations. - Situations where you need to understand - Situations where you need to talk. How well can you participate in these situations? There is no right or wrong answer.

#### Comprehensibility

To improve comprehensibility, items needed to be revised for two main reasons: items appeared to have words with double meanings and items had to be made to be more appropriate to the context. Examples of changes in items are provided in Appendix 3.

#### Comprehensiveness

Overall, the participants found the item pools comprehensive. A few items were suggested to add to the item pool. Most of the suggested topics were already covered within another subdomain, and a few new items were written. More detailed information is provided in Appendix 3.

#### Relevance

Although the relevance of items was not asked in the pilot phase, many participants spontaneously came up with comments on relevance. It was decided to include these comments, which led to deletion of items. The items were deleted because they were 1) not relevant for one of the subgroups of age and 2) irrelevant because of changed contexts (due to COVID-19). Examples of reasons are provided in Appendix 3.

The pilot test resulted in a second version of MyCommunication-Youth. Pool (a) for children consisting of 50 items, pool (b) for adolescents consisting of 69 items and pool (c) for young adults consisting of 72 items.

### Phase 3: Assessing content validity

#### Cognitive interviews in the target population

Twenty-seven children, adolescents and young adults participated (Table 3).

**Table 3:** Participants with communication difficulties in content validity study

Pp	Age	Classification	Gender	Communication problem**	Help reading Y/N***	Multilinguaism?	Included in item analysis?***
P33	17	A	M	Language (DLD)	N	Yes, Dutch/Afghani	Y
C01	25	YA	M	Language (DLD)	N	No	Y
C02*	7	C	M	Language (DLD)	Y	No	Y
C03*	8	C	M	Language (DLD)	N	No	Y
C04	16	A	M	Language (DLD)	N	Yes, Dutch/Lingala	Y
C05	16	A	M	Language (DLD)	N	Yes, Dutch/Arabic	Y
C06	16	A	M	Language (DLD)	N	No	Y
C07	17	A	F	Language (DLD)	N	Yes, Dutch/Chinees	Y
C08	15	A	F	Language (DLD)	N	No	Y
C09	14	A	M	Language (DLD)	N	Yes, Dutch/Arabic	Y
C10	12	C	M	Language (DLD)	N	Yes, Dutch/Polish	Y
C11	16	A	M	Hearing	N	No	Y
C12	15	A	M	Hearing	N	No	Y
C13	17	YA	M	Hearing	N	No	Y
C14	20	YA	M	Hearing	N	No	Y
C15	12	C	F	Speech	N	No	Y
C16	21	YA	M	Speech	N	No	Y
C17	29	YA	M	Speech	N	No	Y
C18	11	C	F	Hearing	N	No	Y
C19	8	C	F	Hearing	N	No	Y
C20	8	C	F	Language (DLD)	Y	No	Y
C21	9	C	M	Language (DLD)	Y	Yes, Dutch/Polish	Y
C22	7	C	M	Language (DLD)	Y	No	N
C23	10	C	F	Language (DLD)	Y	Yes, Dutch/Arabic/English	Y
C24	8	C	F	Language (DLD)	Y	No	Y
C25	5	C	M	Speech/language	Y	No	N
C26	5	C	F	Speech/language	Y	No	N
C27	5	C	M	Speech/language	Y	No	N

*Parent was present during interview

**As reported by participant or parent

***Help reading included reading out loud the entire item or part of the item

****Based on observations of the ability of children to reflect upon communicative participation. If not able, participants were excluded from item analysis

The instruction was found to be comprehensible by most participants. Some participations had trouble understanding the word *situations.* They did however understand what was expected of them. The response options were comprehensible and relevant. Table 4 shows the ratings of the comprehensibility, comprehensiveness and relevance of all three item pools separately and for all pools combined. For the criterium of relevance, we distinguished two types of irrelevant items. 1) Items that contained a relevant participation situation, but contained a communication modality that is not relevant to assess for certain types of communication difficulties; 2) items that contained an irrelevant participation situation.

**Table 4:** Results content validity in target population

Criterium	Children N = 49	Adolescents N = 70	Young adults N = 73	Total unique items N = 106
N items 100% comprehensible	45/49 (91.8%)	69/70 (98.6%)	71/73 (97.3%)	100/106 (94.3%)
N items > 80% comprehensible	48/49 (98%)	70/70 (100%)	73/73 (100%)	106/106 (100%)
N items relevant	35/49 (71.4%)	56/70 (80%)	55/73 (75.3%)	72/106 (67.9%)
Disorder specific item*	* 6/49 (12.2%)*	* 4/70 (5.7%)*	* 15/73 (20.5%)*	* 19/106 (17.9%)*
*Irrelevant participation situation*	* 8/49 (16.3%)*	* 10/70 (12.5%)*	* 3/73 (4.1%)*	* 15/106 (14.1%)*
Comprehensiveness	1 new item suggested	3 new items suggested	0 new items suggested	

*Some items are less relevant to be assessed at specific communication disorders. For example, it is irrelevant to ask a person with speech problems questions about understanding the teacher; even though they do participate in these situations. The situation is relevant, but the item is irrelevant to be assessed when receiving speech and language therapy

#### Comprehensibility

Ratings for comprehensibility of the items were all > 90%, indicating good comprehensibility of the items. Examples of items that were not comprehensible are mentioned in Appendix 4.

#### Relevance

A total of 72/106 unique items were found to be relevant. 19 items were irrelevant for certain subpopulations. These items were mostly about comprehending, or about written language.

A total of 15/106 unique items were found to be irrelevant because of an irrelevant participation situation. This was mostly due to the age of the child, that made the item irrelevant or the item appeared to be cultural specific. Two items obtained bad ratings on both comprehensibility and relevance across all versions. These items were about giving directions and asking for directions. Specific examples are presented in Appendix 4.

#### Comprehensiveness

Many of the suggested items were about daring to tell something. These participants mentioned that for them, confidence plays a big role in their participation. Since these items are about another construct than communicative participation, they were not added.

### Focus group with professionals

Eight professionals participated in this study. Table 5 shows their characteristics.

**Table 5:** Professionals in focus group content validity

Pp	Profession	Work setting	Experience disorder	Child	Adolescent	Young adult	Years work experience
1	SLT	Hospital	Cleft, speech, language, hearing	X	X		30 years
2	SLT	Primary care	Speech, language, voice	X	X	X	7 years
3	SLT	VSO cluster 2	Hearing, language	X	X		8 years
4	SLT	Primary care	Stuttering	X	X	X	30 years
5	SLT and junior researcher	SO cluster 2	Hearing, language	X			7 years
6	Senior researcher	Research institute	Hearing, language	X	X	X	24 years
7	SLT	Primary care	Voice, breathing therapy	X	X	X	16 years
8	SLT	Primary care	Speech, language, hearing, voice	X	X	X	29 years

The preparation task showed that for 26 out of the 120 items, there was less than 85% consensus on one or more criteria on comprehensibility or relevance. These items were discussed in the focus group. In addition, comprehensiveness was discussed per domain. Some items were revised, and on some items, the discussion led to a change of rating. An overview of the final rating on the criteria relevance and comprehensibility can be found in Table 6. An overview of examples of answers of participants can be found in Appendix 4.

**Table 6:** Results focus group on “relevance”, “comprehensibility” and “comprehensiveness”

	Criteria	N items > 85% consensus	N items > 75% consensus	N professionals (%)
1	The item is relevant for measuring communicative participation	113/120 (94%)	118/120 (98%)	
2	The item is relevant for people with communication problems.	117/120 (98%)	118/120 (98%)	
3	The item is relevant to the evaluation of speech and language therapy	106/120 (88%)	116/120 (96%)	
4	The response options are appropriate for measuring the problems a person with communicative participation experiences			8/8 (100%)
6a	The item is clearly and unambiguously worded	118/120 (98%)	120/120 (100%)	
6b	The instruction is clearly and unambiguously worded			8/8 (100%)
7	The response options match the item	120/120 (100%)	120/120 (100%)	

#### Comprehensibility

The instructions were rated as not fully comprehensible by 2/8 participants. The group discussion led to adjustments to the instructions, as presented in Table 2. With this adjustment, the instructions were found to be comprehensible by 8/8 participants. Response options were rated as comprehensible by all participants.

#### Relevance

The main discussion on relevance was about the varying relevance of certain items for measuring/evaluating communicative participation for subpopulations. For example, items that contain written language (texting, emailing) were found to be irrelevant to evaluating communicative participation in people with hearing, speech or voice disorders. For people with language disorders, these items were found to be very relevant. Other topics on relevance are provided in Appendix 4.

#### Comprehensiveness

Eventually, two items were added: *I can participate during a discussion in class; I can tell something to the teacher when I am angry or sad*. Furthermore, the item pool was found to be complete. The new items were pilot-tested and assessed for content validity before they were added to the item pool.

The content validity study led to a final version MyCommunication-Youth, containing MyCommunication-Children of 49 items, MyCommunication-Adolescents of 70 items and MyCommunication-YoungAdults of 73 items.

### Assessment of children’s ability to reflect upon communicative participation

All participants from the age of nine were able to verbalize the meaning of items in their own words (Table 7). Many children aged seven and eight were able to understand the items but were not (always) able to provide an example or verbalize the meaning of the items. The youngest children did not always fully understand the meaning of the items or were not able to link the item to their context. For example, P05 answered the item “I can understand the assignment of the sports teacher” that she was not yet performing a sport because she still had swimming lessons and needed to get her swimming certificate before she could choose a sport. When bringing the item into her context (e.g. interviewer asked her to provide an answer to “I can understand the assignment of the swimming teacher”) she was able to choose an answer and explain why she chose this answer.

**Table 7:** Observations of children’s ability to reflect upon communicative participation

Pp	Age (years)	Communication disorder	Items comprehensible?*	Response format comprehensible?**	Able to reflect on comprehensiveness?
P03	7	Hearing	2	1	No
P04	9	Language	1	1	No
P05	6	Language	2/3	2	No
P08	7	Speech, language	4	3	No
P09	11	Stuttering	1	1	Yes
P11	9	Hearing	1	1	Yes
P12	5	Hearing	3	2	No
P14	12	Language	1	1	Yes
P17	11	Language	1	1	Yes
P18	12	Language	1	1	Yes
P19	9	Speech	1	1	Yes
P20	8	Language	2	2	No
P21	8	Language	2	1	No
P22	7	Language, speech	3	2	No
P23	7	Language	3	3	No
P24	5	Language	3	3	No
CV2	7	Language	1/2	1	No
CV3	8	Language	2	2	No
CV10	12	Language	1	1	Yes
CV15	12	Speech	1	1	Yes
CV18	11	Hearing	1	1	Yes
CV19	8	Hearing	1	1	Yes
CV20	8	Language	1	1	Yes
CV21	9	Language	1	1/2	No
CV22	7	Language	2/3	2	No
CV23	10	Language	1	1	Yes
CV24	8	Language	1	1	Yes
CV25	5	Speech/language	3	3	No
CV26	5	Speech/language	3	3	No
CV27	5	Speech/language	3	3	No

*1 = The child is able to explain the meaning of the items; 2 = The child is unable to explain the meaning of the items, but the researcher subjectively observes that the items are understood; 3 = The child is unable to explain the meaning of the items, and the researcher subjectively observes that the items are partly understood; 4 = The child is unable to explain the meaning of the items, and the researcher subjectively observes that the items are not understood

**1 = Response options understood and explained by participant, 2 = Response options understood, subjectively assessed by researcher, 3 = not understood

One participant (P08) was not able to understand the questions and response options as intended. During the interview, she provided many inconsistent answers. For example, when she had to answer the item “I can have a conversation with my brother or sister”, she responded that she was not able to do so. When rephrasing the question during the interview in two steps (Do you have a brother or sister? – Yes – Can you talk to them? – Yes – what answer would you choose? – Cannot do), it was clear that this type of questioning did not align with her communicative abilities. The response format was comprehensible for all participants, except P08, P23,P24, CV25, CV26 and CV27. Children from about nine years old were able to reflect on comprehensiveness and provided new suggestions for items when necessary.

## Discussion

This paper describes the development of three comprehensive and comprehensible item pools that measure communicative participation in children, adolescents and young adults with various communication problems, and its content validity. The item pools were based on a literature review [12] and two concept elicitation studies [27, 28]. Three final item pools were created: pool (a) for children of primary school age (approximately 5–12) consisting of 49 items, pool (b) for adolescents high school age (approximately 13–18 years old) consisting of 70 items and pool (c) for young adults, working or studying (approximately 19–25 years old) consisting of 73 items.

All items, instructions and response options of the final item pool were found to be comprehensible by the target population and professionals. Most items were found to be relevant to the target population, though some were less relevant for certain subgroups. Overall, the item pools were deemed comprehensive by both the target population and professionals.

The different diagnostic groups found the items relevant to a different extent. Items that were about written communication were irrelevant for people with hearing or speech problems, items that were about comprehension of communication were irrelevant for people with speech problems. As we are working towards an item bank, this should not be a problem. Item banks are a collection of questions from which different measurement instruments can be extracted. For instance, a questionnaire or short form, by choosing an X number of items, or a computer adaptive test. When selecting items for a short form, relevant items can be selected per diagnostic group. This will make it possible to use different questions to measure the same construct on one metric across different diagnosis groups.

This research focuses on children, adolescents, and young adults with various communication problems, including language comprehension issues. Examining comprehensibility was therefore of extra importance. We used guidelines for communication-friendly questionnaires [29] but did not include visualizations. Most individuals with developmental language disorders understood the items, though some preferred visualizations. Further research could explore why visualizations were preferred, as their absence did not cause misinterpretations. The few misunderstood items involved abstract concepts like discussion, conversation, and distress, which are hard to visualize. Literature suggests that while visualizations seem helpful, however, it is unclear why and for whom they work [31]. It would be interesting to further study the added value of visualizations.

Ensuring comprehensiveness was challenging aspect of these studies. We aim to capture a complex, individual-specific construct in a standardized tool. It is always possible to come up with more examples of situations, relevant to certain individuals or groups. However, the construct is considered to be based on a reflective model, and it is therefore not necessary to be fully comprehensive, as each item is considered to reflect the construct. This underscores the importance of meaningful conversations between the SLT and the client after completing the item bank. In these discussions, clients can share additional aspects of communicative participation not captured by the tool, ensuring person-centered care and tailoring therapy to individual needs. In this study, we tried to observe from what age children can reflect upon communicative participation by using the items of the item bank. Most participants could reflect to some extent, except one girl with cerebral palsy and overall developmental delay. Participants aged 5 to 7 often needed more explanation (e.g., clarifying that “sports teacher” included their swimming teacher) or had difficulty understanding response options. For this age group, adjustments in the format (e.g., using dichotomous options as recommended by [19] or administration method (e.g., interviewer administration to help overcome comprehension barriers) are needed.

MyCommunication-Youth is developed for children, adolescents and young adults. Part of the target population in the development of both instruments therefore overlaps with MyCommunication-Adults [14]. Currently, it is unclear whether MyCommunication-Adults or MyCommunication-YoungAdults is more suitable for young adults. Future research should determine which version is most appropriate for young adults. Ideally, the two item banks will be linked, resulting in a single underlying measurement scale. More research is needed to achieve this.

Some limitations of this study are noteworthy. First, as developers of MyCommunication-Youth, we evaluated its content validity, which may have introduced bias. Researchers might have been less critical of responses, and participants might have given socially desirable answers knowing the researchers developed the instrument. To minimize this, respondents were asked to provide examples and justify their answers. However, an independent re-evaluation would be valuable.

Second, not all relevant subpopulations were included. Despite efforts, participants with voice problems were not recruited, affecting the assessment of item pool relevance and comprehensiveness. For individuals with speech problems, items on verbal speaking are crucial, while those on comprehension, reading, and writing are less relevant. We hypothesize that the same applies to people with voice problems.

Third, as researchers, we struggled with eliciting the relevance of items. Since the word relevance is not language level B1, we have asked children “is this item important to ask to other children/adolescents/young adults] with [name communication problem]?”. We changed the wording from relevance to importance, as the difference is subtle difference Dutch. Relevance describes how much something relates to the subject or context, where importance describes how valuable or crucial something is in itself. We have tried to ask for importance and added the subject or context by adding the context of peers with the same communication problem. However, this could still have led to a different interpretation compared to relevance according to COSMIN [25].

## Conclusion

MyCommunication-Youth is developed to measure communicative participation in children, adolescents and young adults with various communication problems. The content validity was assessed in children, adolescents and young adults with speech and language problems and hearing loss. Content validity still needs to be assessed in people with voice disorders, to determine whether this population finds the item pool relevant and comprehensive as well. The next step is to work towards an item bank by assessing the psychometric properties of the three item pools in a large group of children, adolescents and young adults with various communication problems by using item response theory.

## Electronic supplementary material

Below is the link to the electronic supplementary material.

Supplementary Material 1

Supplementary Material 2

## Data Availability

Data will be made available on reasonable request.
