# Supplementary material for: Development and validation of MyCommunication-Youth: A self-report measure for communicative participation in children, adolescents and young adults
Source: J Patient Rep Outcomes. 2025 Jul 10;9:86. doi: 10.1186/s41687-025-00913-1 (PMC12246294; doi:10.1186/s41687-025-00913-1)
Supplement: Supplementary file 1 — Supplementary Material 1 [file 41687_2025_913_MOESM1_ESM.docx]

**Appendix 1: interview guide pilot test**

| **Introduction**   - Thank you for wanting to take part in this study. I'll show you the new questionnaire and I'd love to hear your thoughts. You can say anything, things you like about the questionnaire and things you don’t like about the questionnaire. There are no wrong answers.   **Comprehensibility instruction item bank**   - Can you read the instruction given on the screen. What do you think of the instruction? - Can you tell me what to do next? - Are there any difficult words in it? If so, which words?   **Comprehensibility response options item bank**   - Here you can see an example of a question. There are four response options. Can you read the response options? What would you choose? Why? - What does the word *difficult* means according to you? - If you choose *with a little difficulty*, *with much difficulty* or *cannot do*, you get another question. Would you like to read this question? What would you choose? Why? - Are there any difficult words in it?   **Comprehensibility items (asked per item)**   - Would you like to read the question? - What answer would you give to this question? - Why did you choose this answer? Can you give an example? - Are there any difficult words in it? If so, which words?   - *In case the participant finds the items unclear or difficult:*   How can we make this question easier to understand?   - You choose answer *with a little difficulty/with much difficulty/can't do*, so you get the follow-up question. What would you choose next? - Why did you choose this answer?   **Comprehensiveness per domain**   - You have now seen all questions within the topic [name of topic]. Are you missing any questions within this topic?   **Final questions**   - You've now had all the questions I wanted to show you. Are there any important questions or topics missing? - What do you think of the questionnaire? - Would you like to change anything else about the questionnaire? |
| --- |

**Appendix 2: interview guide content validity**

| **Introduction**   - Thank you for wanting to take part in this study. I'll show you the new questionnaire and I'd love to hear your thoughts. You can say anything, things you like about the questionnaire and things you don’t like about the questionnaire. There are no wrong answers.   **Comprehensibility instruction item bank**   - Can you read the instruction given on the screen. What do you think of the instruction? - Can you tell me what to do next?   **Comprehensibility response options item bank**   - Here you can see an example of a question. There are four response options. Can you read the response options? What would you choose? Why? - What does the word *difficult* means according to you? - If you choose *with a little difficulty*, *with much difficulty* or *cannot do*, you get another question. Would you like to read this question? What would you choose? Why?   **Comprehensibility items (asked per item)**   - Would you like to read the question? - What answer would you give to this question? - Why did you choose this answer? Can you give an example? - Do you think this is an important question to ask people your age with [fill in with communication problem participant] - You choose answer *with a little difficulty/with much difficulty/can't do*, so you get the follow-up question. What would you choose next? - Why did you choose this answer?   **Comprehensiveness per domain**   - You have now seen all questions within the topic [name of topic]. Are you missing any questions within this topic?   **Final questions**   - You've now had all the questions I wanted to show you. Are there any important questions or topics missing? - What do you think of the questionnaire? - Would you like to change anything else about the questionnaire? |
| --- |

**Appendix 3: Examples of changes items pilot test**

**Comprehensibility**

Items were adjusted based on two main reasons:

1. Words with double meanings

Some items of the first version of the item bank needed to be revised, because their wording was misinterpreted. For example in P04: who answered the item “I can introduce myself” as follows:

*“Much difficulty. I am always quickly nervous, sometimes especially when I then have to stand in front of the class. And then you start singing or something.”*

The item "I can introduce myself" was interpreted by P04 as “giving a performance on a school stage”. This is because in the Dutch language, the words *voorstellen* (introducing) and *voorstelling* (performance) are somewhat similar. The use of the word *voorstellen* caused confusion among the youngest group (pool a). This item was adjusted in item pool (a) to: "I can tell someone who I am".

The item “I can tell the teacher that I do not feel well” was unclear for P06:

*“Yes, by not feeling well do you mean being a bit sick or not feeling happy?”*

When answering the question he asked for clarification whether to interpret the item as “feeling sick” or “feeling mentally unhappy”. Both items would be important for him, leading to the proposal to change this item to “I can tell the teacher that I feel sick” and to add an item about feeling mentally unhappy.

1. Fitting items to the context of the target population

In the Dutch language, teachers are called differently on different schools. In primary school teachers are called *juf* (female) of *meester* (male). In high school, teachers are called *leraar* and after high school teachers are called *docent*. We initially started with the items all containing the word *leraar*, but in the item pool (a) this was changed to *juf of meester* and in item pool (c) to *docent.*

The item pool contained multiple items with the word *WhatsAppen.* This word was well interpreted, but some participants noticed that they would call it different: *appen.* One participant (P17) advised to use the word *appen* because as verb you would make it applicable to other apps besides WhatsApp as well:

*“Maybe you should change the word WhatsAppen. I think maybe ‘*appen’ [means Texting in English]*. But it doesn't necessarily have to be with WhatsApp. You can also just do that with iMessage. And some kids use Telegram.”*

The final question of the interview was about the desire to change anything to the item pool. Some participants, all with developmental language disorder, mentioned that they would prefer visual support with the questions. They indicated that visual support is helpful in understanding written text. When assessing the items for comprehensibility, these participants understood the items as intended.

**Comprehensiveness**

1. In the item pool for **children**, two items were added about understanding the explanation and tasks of the gymnastics teacher, and one item was added about asking a group of children if you can play with them.
2. In the item pool for **adolescents**, an item was added about telling the teacher that you feel mentally unhappy. This item was also suggested for the item pool for children and young adults, but eventually not added. For many children, the concept of "mentally unhappy" was not yet clear, so it was not included. Many young adults did not think it was important to talk to the teacher about mental happiness.
3. For **young adults**, one of the participants responded to comprehensiveness as follows:
   *P25: “For me, almost every part of what I do in my life appears in it.”*. No new items were suggested.

**Relevance**

Items were removed based on two main reasons:

1. Item not relevant for the target population

The item pool for children included items that were about using WhatsApp and calling. However, it became evident that children that age do not yet have a phone, so it was decided to remove the items.

The item pool for adolescents contained items about traveling by public transport. It became clear that the majority do not (yet) travel by public transport. It was therefore decided to remove these items from the item pool for adolescents.

1. Changing contexts leading to irrelevant items

The item pools initially included items on online meetings for school and work, based on studies from 2022/2023 when the COVID pandemic made these relevant. However, during the pilot phase, items on online consultations and lessons were found to be irrelevant and were removed.

**Appendix 4: Examples of ratings content validity**

**4.1 Content validity in target population**

**Comprehensibility**

1. Items about talking to strangers

The item pool contained 10 items about talking to strangers. Two of these items appeared to be difficult to comprehend: *Telling an unknown adult what happened* and *Asking a stranger for directions.* For example, CV4 directly asked for explanations after reading the item:

*CV4: “I can tell an unknown adult something about what happened. I don't actually know what they mean by what happened.”*

1. Difficult words

Although all language used in this item pool was language level B1 or lower, some words were still too difficult or abstract to comprehend. For example, words as *discussion, group assignment, emergency* were difficult for a few participants.

CV27: *”Emergency* [Dutch: nood]*, what do you mean with that?”*

*CV24: “I don’t know what you mean by group assignment.”*

**Relevance**

1. Item not relevant, because the child is too young.

A few items were rated as not relevant by the youngest group of children. For example in the items *I can ask the store employee a question* and *I can give a presentation at school*, children understood the item but mentioned that presentations were only given from a particular grade they were not in yet and mentioned that they would always go to the store with their parents.

CV2 responded on the item *I can give a presentation at school: “But we never do that”*

1. Item not relevant, because it contains a cultural specific aspect

Two items appeared to be cultural specific. In the item *I can talk to someone I have a crush on*, one participant mentioned that he was not allowed to have girlfriends according to his religion. The other item was “I can have a conversation with my family during dinner”.

*CV9 responded: “In our culture we have to respect the food. Therefore, we do not talk.”*

1. Item not relevant across all target groups

Two items were rated as not relevant across all versions the item pool. Both items were about directions; one about pointing someone in the right direction and one about asking someone for directions. Participants mentioned that this item was irrelevant, because they would never ask someone for directions and always use Google Maps. This answer was however not consistent. Some participants mentioned that they did find the item relevant, and that it had actually once happened to them that they were lost and had to ask for directions.

**4.2 Content validity in professionals**

Comprehensibility

1. Discussion about response options

However, some participants indicated that they found the step from “with little difficulty” to “with much difficulty” a bit big and would like an additional response option. Others, however, indicated that they found it very clear and did not think that an additional response option was necessary. As none of the people with communication difficulties mentioned this, it was decided not to change the response options.

1. Items not clearly/unambiguously worded

Two items were rated as not clearly/unambiguously worded, which led to minor adjustments. Changes involved minor adjustments (I can conduct an introductory interview/ job interview *at a new workplace* 🡪I can conduct an introductory interview/ job interview *for work*; I can communicate with people *on* a concert or in the theatre 🡪 I can communicate with people *at* a concert or in the theatre).

1. Interpretation of target population

In addition to the adjustments based on the language of the words, some participants commented on some items that they believed their clients would not interpret well. For example in the item *I can have an important conversation with my boss,* one participant found it unclear what was meant by *important conversation*. However, during the interviews with the target population itself (both the pilot test and content validity test), these items were found comprehensible and did not need adaptation.

Relevance

1. Talking in English

The item pool contained several items that were about talking in English (e.g. ordering food/drinks in English). Some of the professionals found these items irrelevant for measuring communicative participation for multiple reasons. One of the professionals worked in a small village where talking in English is not common. Other professionals found it difficult to understand why this could be an important situation and why SLTs would be interested in measuring English proficiency in Dutch clients. Since these items came from the target population itself, we decided to keep the items and report the discrepancy between the target population and the professionals in this paper.

Comprehensiveness

1. Too specific items suggested

During the preparation task, professionals made many suggestions for adding items to the item pool. These suggestions were discussed, and many appeared to be too specific to be included in a generic item bank (i.e. stating what you want at the beautician's). Other suggestions appeared to be important for communicative participation, but the suggested item itself was not measuring communicative participation (i.e. I can specify what I need to have a conversation).
